# Social media addiction in five major mental disorders: a cross-sectional comparative study

**DOI:** 10.1186/s12888-025-07741-z

**Published:** 2026-01-02

**Authors:** Ulaş Korkmaz, Meltem Hazel Şimşek

**Affiliations:** Department of Psychiatry, Giresun University, Giresun, Türkiye

**Keywords:** Social media addiction, Mental disorders, Anxiety, Depression

## Abstract

**Background:**

Social media addiction (SMA) is an increasingly prevalent and significant public health concern. While the relationship between SMA and mental health symptoms, such as anxiety and depression, is well established in the general population, the prevalence, severity, and associations of SMA with mental health symptoms among individuals with mental disorders have not been sufficiently investigated. This study aimed to evaluate the prevalence and severity of SMA, as well as its associations with anxiety and depression symptoms, in diagnostic groups including anxiety disorders (AD), depressive disorders (DD), bipolar disorder (BD), schizophrenia spectrum disorders (SSD), and obsessive-compulsive disorder (OCD).

**Methods:**

A total of 707 patients and 162 healthy controls participated in this cross-sectional comparative study conducted in Türkiye. Participants were assessed using the Sociodemographic Data Form, the Bergen Social Media Addiction Scale (BSMAS), and the Hospital Anxiety and Depression Scale. For the BSMAS, the liberal approach defined the cutoff as obtaining a score of three or more on at least four of the six items, while the conservative approach required a score of three or more on all six items. Group comparisons, correlation analyses, and logistic regression analyses were employed for the statistical analyses.

**Results:**

According to the liberal BSMAS criterion, the prevalence of SMA was highest in the OCD group (44.6%, 95% CI = 35.3% − 54.3%) and lowest in the SSD group (20.8%, 95% CI = 14.5% − 28.9%). Compared to the control group, the OCD group was associated with a higher risk of SMA under both the liberal (OR = 2.45, 95% CI = 1.39–4.31) and conservative (OR = 3.68, 95% CI = 1.58–8.59) criteria. BSMAS scores were significantly higher in the OCD, AD, and DD groups than in the controls. Significant positive correlations were observed between SMA severity and anxiety and depression levels in the AD, DD, and BD groups. In contrast, these associations were not significant in the OCD and SSD groups. Logistic regression analysis showed that when applying the conservative criterion, AD, BD, SSD, and OCD diagnoses could be independent risk factors for SMA.

**Conclusions:**

The findings suggest that major mental disorders may be risk factors for SMA. While co-occurring anxiety and depression symptoms play an important role in this relationship, they only partially explain the association, suggesting that additional mechanisms independent of these symptoms may also be influential, especially in OCD and BD. It is recommended that social media use habits be routinely evaluated in individuals with mental disorders and that comprehensive intervention strategies be developed.

**Clinical trial number:**

Not applicable.

## Background

Social media addiction (SMA) is a behavioral addiction characterized by excessive and uncontrolled use of social media platforms, leading to functional impairment and symptoms such as tolerance, withdrawal, and loss of control. Although initially conceptualized as a subtype of internet addiction, SMA has increasingly been recognized as a distinct condition with validated assessment tools [1, 2].

Today, social media is used by billions of people worldwide, and its usage rates continue to increase [3]. A meta-analysis conducted in 2022 reported the prevalence of SMA in the general population as 17.4% [4]. Another meta-analysis reported that the prevalence of SMA among university students was 18.4%, with this rate increasing to 22.8% in Asian countries [5]. SMA is of critical importance due to its impact on mental health, physical health, and daily life performance [6–8]. The significance of SMA from a mental health perspective lies in its adverse effects on cognitive and emotional functioning. A systematic review and meta-analysis found that SMA was positively associated with symptoms such as anxiety, depression, and loneliness, and negatively associated with self-esteem [9]. This indicates that SMA is a multifaceted problem that should be taken into account in clinical mental health practice.

Studies have indicated that SMA is associated with depressive symptoms [10]. It has also been suggested that individuals with depressive symptoms may increase their risk of addiction by using social media as a coping mechanism or an escape [11]. Anxiety symptoms have also been reported to be closely associated with SMA. One study demonstrated that individuals diagnosed with anxiety disorders (AD) had significantly higher levels of SMA compared to the control group [12]. It has also been reported that intensive and problematic social media use is associated with higher levels of anxiety symptoms [13]. This vicious cycle between SMA and symptoms of anxiety and depression is noteworthy [9, 14]. It has been shown that individuals with severe obsessive-compulsive symptoms use social media more frequently and experience its psychological effects more intensely [15, 16]. In studies investigating the relationship between AD, depressive disorders (DD), and obsessive-compulsive disorder (OCD) with SMA, the samples have mostly consisted of the general population rather than clinical patient groups. Therefore, data regarding prevalence remain limited.

SMA has been investigated only to a limited extent among individuals with severe mental disorders. In individuals with bipolar disorder (BD), it has been reported that the frequency of social media use may vary depending on mood episodes [17]. However, when compared with healthy controls, social media use was not found to be higher [18]. In patients with schizophrenia spectrum and other psychotic disorders (SSD), social media use was found to be similar to that of healthy controls. However, active engagement in sharing on social media was significantly lower than that of healthy controls [19, 20].

In recent years, research on SMA and mental health has increased. The importance of investigating SMA in mental disorders arises both from the additional psychopathological burden that addiction may impose on patients and from its potential consequences on their social functioning. In the literature, SMA has mostly been studied in the general population. However, there is limited evidence regarding the extent of SMA in major mental disorder groups and its association with anxiety and depressive symptoms. Studies conducted with samples largely drawn from the general population and predominantly adolescents, rather than patient groups, may not be sufficient to examine SMA specifically in adult patients with mental disorders. While the association between SMA and subclinical anxiety and depression symptoms has been well established in the general population, there remains a critical lack of studies directly comparing the prevalence and correlates of SMA across well-defined, diagnostically distinct clinical populations. Considering that SMA adversely affects mental, physical, and social health, we believe that investigating SMA in mental disorders is of particular importance.

This study aimed to compare the prevalence and severity of SMA in five major mental disorder groups (AD, DD, BD, SSD, OCD) with those in healthy controls. In addition, it sought to examine the associations between SMA and levels of anxiety and depression within each diagnostic group and to investigate whether mental disorders are associated with a higher risk of SMA.

**Research questions**

1. Do the frequency and severity of SMA differ between patients with mental disorders and healthy controls?
2. Is the presence of a mental disorder diagnosis associated with an increased risk of SMA compared to healthy controls?
3. Within each diagnostic group, is SMA associated with the severity of anxiety and depressive symptoms?

## Methods

### Study design and data collection

This cross-sectional comparative study was conducted at the psychiatry outpatient clinic and the community mental health center of a tertiary hospital in Türkiye. Data were collected using self-report scales completed by both patients diagnosed with one of the five major mental disorders and healthy controls. The study was carried out in accordance with the principles of the Declaration of Helsinki and was approved by the Ethics Committee of Giresun Training and Research Hospital (Decision No: 30.04.2025/07).

The study was conducted between May 2025 and October 2025. A specialist psychiatrist evaluated all participants. Individuals aged 18–65 years who were diagnosed according to DSM-5-TR criteria with DD (major depressive disorder, persistent depressive disorder, and other specified depressive disorder), AD (specific phobia, social anxiety disorder, panic disorder, agoraphobia, generalized anxiety disorder, and unspecified anxiety disorder), OCD, SSD (delusional disorder, brief psychotic disorder, schizophreniform disorder, schizophrenia, schizoaffective disorder, and other specified schizophrenia spectrum and other psychotic disorder), and BD (Type I or II) were included in the study. Individuals with BD and SSD were clinically stable and not in an acute manic or psychotic episode. Similarly, no patients were experiencing severe symptom exacerbations that would impair their ability to complete self-report measures. The control group consisted of individuals with no history of mental disorder who lived in the same region as the patient sample. These participants were selected from hospital staff and volunteers attending the hospital for routine health check-ups. The mental health status of the control group was verified through a brief clinical assessment conducted by a specialist psychiatrist. Informed consent was obtained from all participants. Exclusion criteria for the study population included intellectual disability, the presence of neurological disorders, comorbid mental disorders, being in an acute psychotic or manic episode, and having a clinical condition that would prevent the completion of the scales.

All eligible patients who met the inclusion criteria and were consecutively admitted to the psychiatry outpatient clinic and community mental health center during the study period were invited to participate. Healthy controls were recruited using a convenience sampling approach from hospital staff and individuals undergoing routine health check-ups at the same institution and within the same geographical region. Therefore, this study does not represent a total population sample but rather a consecutively recruited clinical sample.

### Measures

#### Sociodemographic data form

Developed by the researchers, this form contained basic sociodemographic information such as participants’ age, gender, marital status, educational level, and employment status.

#### Bergen Social Media Addiction Scale (BSMAS)

This scale measures the level of addiction related to individuals’ social media use. It was developed by Andreassen et al. [21, 22], and its Turkish validity and reliability were established by Demirci [2]. The scale is a 6-item, 5-point Likert-type (1 = very rarely, 5 = very often) self-report instrument designed to assess the core components of SMA: salience, tolerance, mood modification, withdrawal, conflict, and relapse. The total score ranges from 6 to 30, with higher scores indicating a greater risk of SMA. Two different approaches are used in determining the cutoff: in the liberal approach, a score of three or more on at least four of the six items is required, whereas in the conservative approach, a score of three or more on all six items is required. In this study, analyses were conducted according to both approaches. The use of both liberal and conservative cut-off criteria is consistent with previous studies employing BSMAS [21, 23, 24]. The liberal approach allows for the identification of individuals at risk for problematic social media use, whereas the conservative approach captures more severe and clinically relevant levels of addiction, thereby providing a broader and more nuanced assessment of SMA.

#### Hospital Anxiety and Depression Scale (HADS)

This scale was developed by Zigmond and Snaith [25], and its Turkish validity and reliability were established by Aydemir et al. [26]. It is a 14-item, 4-point Likert-type (scored between 0 and 3) self-report instrument designed to assess individuals’ anxiety and depression symptoms over the past week. The scale consists of two subscales: Anxiety (7 items) and Depression (7 items). Scores for each subscale range from 0 to 21, with higher scores indicating greater symptom severity in the respective subscale.

### Statistical analysis

The statistical analyses were conducted using IBM SPSS Statistics version 27.0. Skewness and kurtosis values within the range of ± 1.5 were considered indicative of normal distribution [27]. Descriptive statistics (mean, standard deviation, median, interquartile range, frequency) were calculated for sociodemographic and clinical variables. For group comparisons of continuous variables, one-way analysis of variance (ANOVA) or the Kruskal–Wallis H test was used, depending on the distribution. Categorical variables were analyzed using the chi-square test. To compare BSMAS, HADS-Anxiety, and HADS-Depression scores across diagnostic groups, multivariate analysis of variance (MANOVA) and multivariate analysis of covariance (MANCOVA) were applied. Age, education level, gender, marital status, and employment status were included as covariates in the model. For significant results, post-hoc comparisons were performed using Tukey HSD (for ANOVA with homogeneity of variances), Games-Howell (for ANOVA with unequal variances), or Bonferroni tests (for MANOVA/MANCOVA and chi-square tests). The relationships between BSMAS and HADS scores were assessed using Pearson’s correlation coefficient. In addition, to predict SMA risk, binary logistic regression analyses were conducted while controlling for sociodemographic variables. For each variable, regression coefficients (b), odds ratios (OR), and 95% confidence intervals (CI) were reported. A p-value < 0.05 was considered statistically significant.

## Results

The study sample consisted of 869 participants. In the total sample, the mean age was 36.94 ± 11.96 years, and the mean duration of education was 11.91 ± 3.91 years. Regarding gender, 57.4% were female, and 42.6% were male. In terms of marital status, 51.6% were single, and 48.4% were married. Concerning employment status, 41.4% of participants were employed, while 58.6% were unemployed. The distribution of sociodemographic data according to groups and the comparisons between groups are presented in Table 1.

Table 2 presents the prevalence of SMA by diagnostic groups and the between-group comparisons according to the liberal criterion of the BSMAS (a score of three or more on at least four of the six items) and the conservative criterion (a score of three or more on all six items). According to the liberal criterion of the BSMAS, SMA rates differed significantly across diagnostic groups (X² = 20.360, *p* < 0.001). Post-hoc analyses showed that the OCD group had a significantly higher prevalence of SMA than all other groups (all *p* < 0.05). No significant differences were observed among the AD, DD, BD, SSD, and control groups after Bonferroni correction. Although the difference between the AD group and the control group was not significant after Bonferroni correction, pairwise comparisons revealed a higher SMA rate in the AD group (X² = 4.614, *p* = 0.032). A similar trend was observed according to the conservative criterion (X² = 13.028, *p* = 0.023). Again, after Bonferroni correction, the differences between the AD and BD groups compared to controls were not significant. However, pairwise comparisons showed that SMA rates were higher in the AD group (X² = 5.455, *p* = 0.020) and the BD group (X² = 5.774, *p* = 0.016) than in controls.

Table 3 compares the group means of BSMAS, HADS-Anxiety, and HADS-Depression scores. BSMAS scores differed significantly across groups (F = 8.711, *p* < 0.001). Post-hoc analyses showed that the OCD group had significantly higher BSMAS scores than all other groups (all *p* < 0.05). The AD and DD groups also had significantly higher BSMAS scores than the control and SSD groups (*p* < 0.05), while BSMAS scores in the BD group did not differ significantly from those of the control group. The SSD group had significantly lower BSMAS scores than the control group (*p* < 0.05). Similarly, HADS-Anxiety scores also differed significantly across groups (F = 49.451, *p* < 0.001). Post-hoc tests revealed that the AD, DD, and OCD groups had significantly higher anxiety scores than the SSD and BD groups, and all patient groups scored significantly higher than the control group (all *p* < 0.05). Similarly, HADS-Depression scores showed significant group differences (F = 33.177, *p* < 0.001). The DD group had significantly higher depression scores than all other groups, followed by the AD group. Scores in the SSD, BD, and OCD groups did not differ significantly from one another, but all patient groups scored significantly higher than the control group (all *p* < 0.05). In the MANCOVA analysis, with age, education level, gender, marital status, and employment status included as covariates, BSMAS scores were significantly higher in the AD group than in the control group, whereas no other group differences were significant (F = 3.202, *p* = 0.007). For HADS-Anxiety (F = 31.087, *p* < 0.001) and HADS-Depression (F = 23.346, *p* < 0.001), the ranking pattern remained largely unchanged.

Pearson correlation coefficients between BSMAS scores and HADS-Anxiety and HADS-Depression scores are presented in Table 4. Significant correlations were found between BSMAS scores and both HADS-Anxiety and HADS-Depression scores in the control, AD, DD, and BD groups. Notably, no significant correlations were observed between BSMAS scores and either anxiety or depression levels in the OCD and SSD groups, distinguishing these groups from the other diagnostic categories.

**Table 1:** Sociodemographic characteristics of participants by group

Mean ± SD / Median (Q1-Q3) / *n* (%)	Control (*n* = 162)	AD (*n* = 210)	DD (*n* = 162)	BD (*n* = 114)	SSD (*n* = 120)	OCD (*n* = 101)	Statistic	*p*
**Age (years)**	34.14 ± 9.28	34.87 ± 12.74	36.92 ± 12.73	41.38 ± 11.13	43.28 ± 10.64	33.26 ± 11.06	F = 16.141	< 0.001
**Education (years)**	15 (12–16)	12 (10–15)	12 (8–14)	11 (8–13)	11 (5.25-12)	12 (12–26)	H = 124.757	< 0.001
**Gender**							X² = 64.585	< 0.001
Male	74 (45.7%)	79 (37.6%)	44 (27.2%)	56 (49.1%)	85 (70.8%)	32 (31.7%)		
Female	88 (54.3%)	131 (62.4%)	118 (72.8%)	58 (50.9%)	35 (29.2%)	69 (68.3%)		
**Marital status**							X² = 30.062	< 0.001
Single	76 (46.9%)	99 (47.1%)	74 (45.7%)	60 (52.6%)	89 (74.2%)	50 (49.5%)		
Married	86 (53.1%)	111 (52.9%)	88 (54.3%)	54 (47.4%)	31 (25.8%)	51 (50.5%)		
**Employment status**							X² = 161.313	< 0.001
Employed	133 (82.1%)	81 (38.6%)	64 (39.5%)	32 (28.1%)	16 (13.3%)	34 (33.7%)		
Unemployed	29 (17.9%)	129 (61.4%)	98 (60.5%)	82 (71.9%)	104 (86.7%)	67 (66.3%)		

n = Sample size; p = Statistical significance level; Q1 = First quartile; Q3 = Third quartile; SD = Standard deviation; F = ANOVA test statistic; H = Kruskal–Wallis H test statistic; X² = Chi-square test statistic

AD: Anxiety disorders, DD: Depressive disorders, BD: Bipolar disorder, SSD: Schizophrenia spectrum and other psychotic disorders, OCD: Obsessive-compulsive disorder

**Table 2:** Prevalence of social media addiction according to the Liberal (4/6) and conservative (6/6) criteria of the Bergen Social Media addiction scale

*n* (%)	Controlᵃ (*n* = 162)	ADᵇ (*n* = 210)	DDᶜ (*n* = 162)	BDᵈ (*n* = 114)	SSD^e^ (*n* = 120)	OCD^f^ (*n* = 101)	X²	*p*	Post-hoc
**BSMAS 4/6**							20.360	< 0.001	f > b = c = d = e = a
Yes	41 (25.3%)	75 (35.7%)	50 (30.9%)	30 (26.3%)	25 (20.8%)	45 (44.6%)			
No	121 (74.7%)	135 (64.3%)	112 (69.1%)	84 (73.7%)	95 (79.2%)	56 (55.4%)			
**BSMAS 6/6**							13.028	0.023	f > b = c = d = e = a
Yes	10 (6.2%)	30 (14.3%)	17 (10.5%)	18 (15.8%)	15 (12.5%)	20 (19.8%)			
No	152 (93.8%)	180 (85.7%)	145 (89.5%)	96 (84.2%)	105 (87.5%)	81 (80.1%)			

n = Sample size; p = Statistical significance level; X² = Chi-square test statistic

BSMAS: Bergen social media addiction scale, AD: Anxiety disorders, DD: Depressive disorders, BD: Bipolar disorder, SSD: Schizophrenia spectrum and other psychotic disorders, OCD: Obsessive-compulsive disorder

**Table 3:** Social media addiction, anxiety, and depression scores by groups

Mean ± SD	Controlᵃ (*n* = 162)	ADᵇ (*n* = 210)	DDᶜ (*n* = 162)	BDᵈ (*n* = 114)	SSD^e^ (*n* = 120)	OCD^f^ (*n* = 101)	F	*p*	Post-hoc
BSMAS	12.33 ± 5.42	14.38 ± 6.40	13.87 ± 6.29	12.60 ± 4.47	10.65 ± 5.44	15.92 ± 6.70	8.711	< 0.001	f > b = c > a = d > e
HADS-Anxiety	6.10 ± 4.10	12.17 ± 4.67	12.16 ± 4.46	7.81 ± 5.15	8.75 ± 4.50	11.49 ± 4.66	49.451	< 0.001	b = c = f > e = d > a
HADS-Depression	5.39 ± 3.63	9.45 ± 4.31	11.33 ± 4.59	7.63 ± 4.62	9.11 ± 4.31	8.82 ± 4.86	33.177	< 0.001	c > b > e = d = f > a

n = Sample size; p = Statistical significance level; SD = Standard deviation; F = univariate test statistic (following MANOVA)

BSMAS: Bergen social media addiction scale, HADS: Hospital anxiety and depression scale, AD: Anxiety disorders, DD: Depressive disorders, BD: Bipolar disorder, SSD: Schizophrenia spectrum and other psychotic disorders, OCD: Obsessive-compulsive disorder

**Table 4:** Correlations between the Bergen Social Media Addiction Scale (BSMAS) and HADS-Anxiety and HADS-Depression scores

	Control (*n* = 162)	AD (*n* = 210)	DD (*n* = 162)	BD (*n* = 114)	SSD (*n* = 120)	OCD (*n* = 101)
HADS-Anxiety r: p:	0.278 < 0.001	0.310 < 0.001	0.416 < 0.001	0.285 0.004	0.107 0.320	0.117 0.250
HADS-Depression r: p:	0.192 0.015	0.285 < 0.001	0.240 0.003	0.208 0.036	0.084 0.434	0.115 0.262

n = Sample size; p = Statistical significance level; r = Correlation coefficient

HADS: Hospital anxiety and depression scale, AD: Anxiety disorders, DD: Depressive disorders, BD: Bipolar disorder, SSD: Schizophrenia spectrum and other psychotic disorders, OCD: Obsessive-compulsive disorder

The results of the binary logistic regression analysis, adjusted for age, education level, gender, marital status, and employment status as covariates, are presented in Table 5. According to the liberal criterion of the BSMAS, compared to the control group, the OCD group had a 2.45-fold higher risk of SMA (95% CI = 1.393–4.308), while the AD group had a 1.72-fold higher risk (95% CI = 1.057–2.808). The increased risks observed in the other three groups were not statistically significant. When anxiety and depression scores were added to the model, the predictive effects for the OCD and AD groups under the liberal criterion lost their significance. When the conservative criterion of the scale was applied, the odds ratios associated with SMA increased markedly: BD (OR = 4.15, 95% CI = 1.700–10.142), SSD (OR = 3.47, 95% CI = 1.269–9.477), OCD (OR = 3.68, 95% CI = 1.580–8.587), and AD (OR = 2.50, 95% CI = 1.140–5.489). When anxiety and depression scores were again included in the model, the predictive effects for the BD group (b = 1.172, OR = 3.230, 95% CI = 1.281–8.144) and the OCD group (b = 0.905, OR = 2.473, 95% CI = 1.015–6.026) remained significant.

**Table 5:** Logistic regression analysis of social media addiction risk according to the criteria of the Bergen Social Media Addiction Scale

Reference: Control	SMA present (1) or absent (0) according to 4/6 criterion			SMA present (1) or absent (0) according to 6/6 criterion		
	b	OR	95% CI	b	OR	95% CI
AD	0.544	1.722	1.057–2.808*	0.917	2.502	1.140–5.489*
DD	0.382	1.465	0.864–2.486	0.671	1.957	0.828–4.624
BD	0.432	1.540	0.832–2.851	1.424	4.152	1.700-10.142*
SSD	0.075	1.077	0.527–2.201	1.244	3.469	1.269–9.477*
OCD	0.896	2.450	1.393–4.308*	1.304	3.684	1.580–8.587*

Controlled for age, education level, gender, marital status, and employment status

* *p* < 0.001

b = Unstandardized beta coefficient; OR = Odds ratio; CI = Confidence interval

SMA: Social media addiction, AD: Anxiety disorders, DD: Depressive disorders, BD: Bipolar disorder, SSD: Schizophrenia spectrum and other psychotic disorders, OCD: Obsessive-compulsive disorder

## Discussion

In this study, the prevalence and severity of SMA were investigated in five major mental disorder groups (AD, DD, BD, SSD, OCD) and a healthy control group. The relationship between SMA and anxiety and depression levels was examined, and it was also analyzed whether having any mental disorder was associated with an increased risk of SMA compared to the control group. The results showed that the prevalence and severity of SMA differed significantly across diagnostic groups, with the highest rates observed in individuals with OCD. Regression analyses indicated that OCD and BD were independently associated with SMA, even after controlling for anxiety and depression symptoms. In addition, while SMA severity was positively associated with anxiety and depression in most groups, no such associations were observed in the OCD and SSD groups.

According to the liberal criterion of the BSMAS, the prevalence of SMA was 25.3% in the healthy control group, 35.7% in the AD group, 30.9% in the DD group, 26.3% in the BD group, 20.8% in the SSD group, and 44.6% in the OCD group. According to the conservative criterion, SMA prevalence was 6.2% in the healthy control group, 14.3% in the AD group, 10.5% in the DD group, 15.8% in the BD group, 12.5% in the SSD group, and 19.8% in the OCD group. The discrepancy reflects differences in cut-off stringency. The liberal criterion captures broader problematic use, resulting in higher prevalence across all groups, including controls. The conservative criterion identifies more severe SMA. Studies indicate that the rate of SMA in the general population may range between 5% and 25% [4, 23, 28]. The SMA rate observed in the healthy controls in the present study is consistent with previously reported population-based rates. Similarly, the association between problematic social media use and levels of depression and anxiety in healthy controls [13] was also confirmed in this study. These prevalence findings indicate that SMA represents a clinically relevant issue in individuals with mental disorders, particularly in OCD and AD. The substantially higher rates observed in these groups highlight the need for increased clinical awareness and routine assessment of problematic social media use in psychiatric settings.

All mental disorder groups except SSD exhibited higher SMA rates than the control group. However, statistical analyses showed that the OCD group exhibited a higher rate of SMA than the other groups. Moreover, the OCD group had significantly higher BSMAS scores than all other groups. Under both the liberal and conservative criteria, the OCD group showed a stronger association with SMA risk than the control group, and under the conservative criterion, this association remained significant even after controlling for anxiety and depression. Previous literature has identified positive correlations between problematic social media use and obsessive-compulsive symptoms [22, 29]. In particular, fear of missing out and compulsive social media use have been reported to play a role in the relationship between obsessive-compulsive symptoms and intensive social media use [30]. Considering that perfectionism is associated with both fear of missing out [31] and OCD [32], this effect can be better understood. Moreover, obsessions in OCD may drive individuals toward persistent searching and checking behaviors on social media.

On the other hand, no significant correlation was found between BSMAS and the HADS subscales in the OCD group, and the group difference was no longer significant in the MANCOVA analysis. This lack of association suggests that the elevated SMA observed in OCD is not primarily driven by concurrent anxiety or depressive symptom severity. Rather, the persistence of SMA-related behaviors in OCD may reflect disorder-specific cognitive and behavioral mechanisms, which are not adequately captured by general measures of anxiety and depression. Additionally, these findings suggest that other factors may contribute to the relationship between OCD and SMA, which may, for example, be moderated by sociodemographic variables.

No significant difference in SMA prevalence was observed between the control group and any patient group other than OCD. One possible reason for this may be the large number of groups. Indeed, before correcting for multiple comparisons, the AD group had a higher SMA rate than the control group. Moreover, in the MANCOVA analysis, BSMAS scores were significantly higher in the AD group compared to controls. In the AD group, BSMAS scores were positively correlated with levels of anxiety and depression, and regression analysis demonstrated a predictive effect on SMA. This effect was valid for both the liberal and conservative criterion models. Numerous reviews and meta-analyses have identified positive correlations between SMA and anxiety symptoms [33, 34]. In a study conducted with a clinical population, individuals with AD were found to be more dependent on social media compared to the control group [12]. Variables such as sleep deprivation, social comparison, and feedback-seeking behaviors have been reported to influence the relationship between SMA and anxiety symptoms [35]. Factors such as insecure attachment have also been reported to strengthen this association [36]. SMA may contribute to increased depression, anxiety, and stress, particularly in adolescents, by impairing attention and cognitive control [37]. In addition, problematic social media use may serve as a maladaptive emotion regulation strategy [38]. In the regression analysis, when anxiety and depression levels were controlled for, the predictive effect of the AD group lost its significance. This finding is consistent with the literature on the relationship between anxiety, depression, and SMA.

The SMA prevalence in the DD group was similar to that of the other groups, except OCD. However, the DD group had higher BSMAS scores than the control group. In addition, positive correlations were found between BSMAS scores and both anxiety and depression scores in this group. SMA has been shown to be significantly associated with depression [39]. Factors such as social comparisons [40], insomnia [41], and insufficient social support [42] may play a role in this relationship. Social media use is also frequently employed as a means of coping with psychological distress [43]. Difficulties in emotion regulation may serve as an important mechanism in the connection between depression and SMA [44]. Furthermore, metacognitive processes may also represent a risk factor for SMA [45]. The finding that the DD group was not a significant predictor of SMA, despite its significantly higher BSMAS scores relative to controls, may be explained by symptoms such as anhedonia or psychomotor retardation in individuals with DD. However, the large number of groups should also be taken into consideration. Indeed, one study reported that the DD group obtained higher BSMAS scores than the control group [46]. Additionally, a study conducted in Türkiye found that patients diagnosed with DD were more dependent on social media compared to controls [12].

When evaluated according to the conservative criteria, the BD group was found to have a higher prevalence of SMA compared to the control group. However, there was no significant difference in terms of BSMAS scores. In the BD group, BSMAS scores were correlated with anxiety and depression scores. In the regression analysis, the BD group was a predictor in the conservative criterion model, and this predictive effect remained significant even after anxiety and depression scores were included in the model. This suggests that specific BD-related characteristics beyond anxiety and depression may contribute to intensive social media use. For example, impulsivity may represent a shared mechanism between BD and SMA [47, 48]. Although the literature on SMA in BD is limited, a study conducted among adolescents demonstrated a significant positive association between screen time and manic symptoms in subsequent years [49]. Although social media engagement may vary across depressive, euthymic, and hypomanic states, mood episode status was not systematically assessed in the present study, as patients in acute manic episodes were excluded. Therefore, the observed association likely reflects more stable BD-related traits rather than state-dependent effects. Further research is needed to determine the specific features of the relationship between BD and SMA.

The SMA prevalence in the SSD group was similar to that of the control group. However, BSMAS scores were lower than those of controls. This finding may be partly attributable to the negative symptoms prevalent in SSD. Indeed, Rekhi et al. [50] found a negative correlation between negative symptoms and social media use. This pattern may also reflect the differential roles of negative and positive symptoms in social media use among individuals with SSD. Negative symptoms such as avolition, anhedonia, and social withdrawal may limit engagement with social media platforms, resulting in lower overall use and addiction severity. In contrast, positive symptoms, including suspiciousness and altered self-other boundaries, may lead certain individuals to use social media in a more maladaptive or excessive manner. However, as symptom dimensions were not assessed separately in this study, this distinction remains speculative and should be examined in future research using symptom-specific measures.

In this study, no significant associations were observed in the correlation analyses for the SSD group. In the conservative criterion regression model, however, SSD showed a significant predictive effect until control variables were added to the model. This finding may suggest that SSD could be a risk factor for excessive social media use, although this effect appears to be moderated by levels of depression and anxiety. Previous studies have reported that social media may blur self–other boundaries, thereby increasing delusional thinking and potentially exacerbating psychotic symptoms [51]. Nevertheless, research on SMA in SSD populations remains quite limited, and further studies are warranted.

Another finding was that in the regression analysis using the conservative criterion, the predictive effects of mental disorders were stronger than in the model using the liberal criterion. The differences observed between the liberal and conservative BSMAS criteria may reflect the distinct severity thresholds captured by these two approaches. The liberal criterion identifies individuals at risk for problematic or emerging social media use, whereas the conservative criterion is more likely to detect cases with more persistent, clinically relevant addictive behaviors. Accordingly, associations with psychiatric diagnoses became more pronounced under the conservative criterion, suggesting that mental disorders may be more strongly linked to more severe forms of SMA rather than milder or subthreshold patterns.

Considering that SMA can negatively affect mental health, physical health, and daily functioning performance [6–8], the findings of this study highlight the importance of assessing social media use in individuals with mental disorders. When necessary, psychoeducation-based interventions may be implemented. Cognitive-behavioral therapies can be applied for SMA [52]. However, currently no evidence exists for the effectiveness of cognitive-behavioral therapies in individuals with comorbid mental disorders and SMA. Moreover, the positive correlation between SMA and anxiety and depression highlights the necessity of a holistic approach in patients with accompanying mental health symptoms. From a clinical perspective, the findings suggest that social media use should be routinely assessed in psychiatric practice, particularly among patients diagnosed with OCD and AD, who demonstrated the highest risk for SMA. Clinicians should be alert to excessive or compulsive social media use and consider incorporating psychoeducation on healthy digital habits into standard treatment plans. In addition, behavioral activation strategies and interventions targeting maladaptive coping mechanisms may be beneficial, especially in patients whose social media use is associated with anxiety and depressive symptoms. Integrating SMA-related assessment and interventions into routine care may help reduce functional impairment and improve overall treatment outcomes.

Studies investigating the relationship between SMA and mental health symptoms have mostly been conducted in the general population and in younger age groups. The strengths of our study include the simultaneous evaluation of five major disorder groups and the inclusion of individuals aged 18–65 years. On the other hand, the study has several limitations. The cross-sectional design precludes any conclusions regarding causal relationships between SMA and psychiatric symptoms. All psychological measures were based on self-report instruments, which are inherently subject to recall bias, response bias, and social desirability effects. Recruitment from a single center may limit the generalizability of the findings to broader and more diverse populations. The selection of the control group from hospital staff and volunteers may introduce selection bias and increase the likelihood of social desirability bias. Heterogeneity within diagnostic groups may have introduced variability in clinical characteristics, which could have influenced the observed associations. The lack of disorder-specific scales for BD, SSD, and OCD may have limited the sensitivity of the assessments in capturing disorder-specific symptom profiles. Structured diagnostic interviews (e.g., MINI, SCID, SCAN, or CIDI) were not administered, and diagnoses were based on routine clinical assessments conducted by specialist psychiatrists according to DSM-5-TR criteria, which may have affected the diagnostic precision of the clinical assessments. Although the BSMAS is a widely used and psychometrically validated instrument, it is a brief screening tool for addictive patterns of social media use. As such, it may not fully capture the complexity, contextual features, and functional impacts of SMA, including platform-specific behaviors, motivational aspects, and usage patterns over time. Finally, the study did not collect data on the specific social media platforms used by participants. Different platforms may be associated with distinct patterns of engagement, social comparison, compulsivity, or emotional reactivity.

## Conclusion

The results of this study demonstrate that individuals diagnosed with mental disorders are at high risk for SMA. In particular, patients diagnosed with OCD and AD showed markedly higher prevalence and severity of SMA, while the severity of anxiety and depression was also strongly associated with SMA. These findings emphasize the importance of considering social media use in mental health services. In clinical practice, assessing social media use habits and potential addiction symptoms in psychiatric patients, as well as developing preventive and interventional strategies, are of public health significance. Future research should prioritize longitudinal designs to clarify the temporal and causal relationships between SMA and psychiatric symptoms across different diagnostic groups. Such studies would help disentangle whether excessive social media use represents a contributing factor, a consequence, or a maintaining mechanism of psychopathology. In parallel, the development and evaluation of targeted, diagnosis-sensitive intervention strategies addressing problematic social media use may represent an important next step for improving clinical outcomes and psychosocial functioning in individuals with mental disorders.

## Data Availability

The datasets used during the current study are available from the corresponding author on reasonable request.

## Abbreviations

SMA: Social media addiction
AD: Anxiety disorders
DD: Depressive disorders
BD: Bipolar disorder
SSD: Schizophrenia spectrum disorders
OCD: Obsessive-compulsive disorder
DSM-5-TR: Diagnostic and Statistical Manual of Mental Disorders, Fifth Edition, Text Revision
BSMAS: Bergen Social Media Addiction Scale
HADS: Hospital Anxiety and Depression Scale
SPSS: Statistical Package for the Social Sciences
ANOVA: One-way analysis of variance
MANOVA: Multivariate analysis of variance
MANCOVA: Multivariate analysis of covariance
OR: Odds ratio
CI: Confidence interval
SD: Standard deviation
